# An inbred line of the diploid strawberry *Fragaria vesca *f. *semperflorens *for genomic and molecular genetic studies in the Rosaceae

**DOI:** 10.1186/1746-4811-5-15

**Published:** 2009-10-31

**Authors:** Janet P Slovin, Kyle Schmitt, Kevin M Folta

**Affiliations:** 1 Genetic Improvement of Fruits and Vegetables Laboratory, U.S. Department of Agriculture - Agricultural Research Service, Henry A Wallace Beltsville Agricultural Research Center, 10300 Baltimore Avenue, Beltsville, MD 20705, USA; 2 Horticultural Sciences Department and the Graduate Program in Plant Molecular and Cellular Biology, 1301 Fifield Hall, University of Florida, Gainesville, FL 32611, USA

## Abstract

**Background:**

The diploid woodland strawberry (*Fragaria vesca*) is an attractive system for functional genomics studies. Its small stature, fast regeneration time, efficient transformability and small genome size, together with substantial EST and genomic sequence resources make it an ideal reference plant for *Fragaria *and other herbaceous perennials. Most importantly, this species shares gene sequence similarity and genomic microcolinearity with other members of the Rosaceae family, including large-statured tree crops (such as apple, peach and cherry), and brambles and roses as well as with the cultivated octoploid strawberry, *F*. ×*ananassa*. *F. vesca *may be used to quickly address questions of gene function relevant to these valuable crop species. Although some *F. vesca *lines have been shown to be substantially homozygous, in our hands plants in purportedly homozygous populations exhibited a range of morphological and physiological variation, confounding phenotypic analyses. We also found the genotype of a named variety, thought to be well-characterized and even sold commercially, to be in question. An easy to grow, standardized, inbred diploid *Fragaria *line with documented genotype that is available to all members of the research community will facilitate comparison of results among laboratories and provide the research community with a necessary tool for functionally testing the large amount of sequence data that will soon be available for peach, apple, and strawberry.

**Results:**

A highly inbred line, YW5AF7, of a diploid strawberry *Fragaria vesca *f. *semperflorens *line called "Yellow Wonder" (Y2) was developed and examined. Botanical descriptors were assessed for morphological characterization of this genotype. The plant line was found to be rapidly transformable using established techniques and media formulations.

**Conclusion:**

The development of the documented YW5AF7 line provides an important tool for Rosaceae functional genomic analyses. These day-neutral plants have a small genome, a seed to seed cycle of 3.0 - 3.5 months, and produce fruit in 7.5 cm pots in a growth chamber. YW5AF7 is runnerless and therefore easy to maintain in the greenhouse, forms abundant branch crowns for vegetative propagation, and produces highly aromatic yellow fruit throughout the year in the greenhouse. *F. vesca *can be transformed with *Agrobacterium tumefaciens*, making these plants suitable for insertional mutagenesis, RNAi and overexpression studies that can be compared against a stable baseline of phenotypic descriptors and can be readily genetically substantiated.

## Background

The family Rosaceae is comprised of diverse fruit, nut and ornamental plants. At this time, resources that will accelerate research efforts in this important crop family are being developed [1]. Genomes from three family members (peach, apple, and strawberry) are currently being sequenced and a massive number of transcribed sequences are being catalogued. While the amount of structural genomics information is increasing, the ability to put this information to work in a functional genomics context has not significantly advanced across rosaceous species. The growing wealth of genomics-level information requires development of agile transformation systems to enable direct tests of gene function. Unfortunately, the majority of the valuable crops in this family are large-architectured tree crops with long juvenility periods and substantial space requirements. Brambles and roses are challenging in culture, and require substantial time for regeneration. These characteristics slow the speed of discovery and greatly decrease the practicality of gene function studies in these systems. However, analysis of genome structure and content indicate remarkable similarities in protein sequence and colinearity between the studied members of the family [2], suggesting that gene regulation and function may be highly translatable between species.

An excellent candidate system that circumvents many of these problems is the diploid strawberry (*Fragaria vesca *L.; 2n = 14). *F. vesca *possesses many attributes that make it ideal for genomics, either as a reverse or forward genetic system or as a rapid means for direct tests of gene function [3-5]. It is a small plant commonly found along the edges of woodlands, with a wide distribution throughout Europe, Asia, and the United States [6]. The wild everbearing, or day-neutral form, *F. vesca *f. *semperflorens*, is native to Europe [6], and produces fruit throughout the year in the greenhouse. Forms exist that reproduce by seed or branch crowns only, and there exist forms that are capable of reproducing vegetatively by runners as well [7]. The runnerless type, called Bush Alpine or Gaillon strawberry, tends to bear larger fruit than the runnered type [6]. White or pink flowered forms, single and double flowered forms, white or red fruited forms, as well as forms with three leaflets or one leaflet, have been described by Richardson [8-11]. Fruit aromas of lab-grown genotypes range from grape-like to sweet overripe banana and pineapple (Slovin, personal observation). Importantly, substantial evidence indicates that *F. vesca *shares a common ancestor with at least one of the subgenomes within the commercial octoploid strawberry, *F*. ×*ananassa *[12,13], making findings in the reference species relevant to the cultivated germplasm.

There are additional attributes that make *F. vesca *an attractive system to answer basic biological questions as well as solve agriculturally important problems. *F. vesca *is rapidly regenerable from tissue culture and can be transformed using *Agrobacterium tumefaciens *[14-17]. Each plant produces many achenes, making it suitable for genetic studies. The seed to seed cycle of *F. vesca *is complete in less than 4 months, and the plant can be grown to seed in a 7.5-10 cm pot in a small greenhouse, or even on a lighted laboratory shelf. Approximately 1% of the genome sequence appears in public databases (GenBank numbers EU024823-EU024872), the most of any Roscaceae family member [1]. The genome of these plants is small, approximately 200 Mb [18-20]. The genome of *F. vesca *line 'Hawaii-4' (accession PI551572) is currently being sequenced and a substantial body of sequences from transcribed genes of octoploid and diploid *Fragaria *species is available.

**Figure 1:**
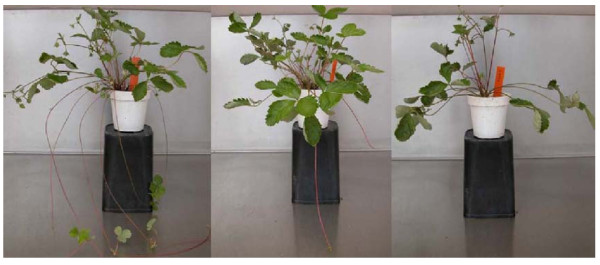
**A sample of phenotypic variation observed among *F. vesca *lines**. Thirty-seven F4 inbred plants of accession Hawaii-4 were grown under uniform greenhouse conditions. While most plants maintained similar appearance (center) extreme phenotypes were still observed (left and right). These plants exhibited substantial differences in runnering and flowering time.

One issue that made *F. vesca *less than optimal for genome function studies is that, although being self-fertile and therefore likely to be substantially inbred, individual plants in populations of various lines growing under essentially uniform conditions in the greenhouse or growth chamber exhibited substantial phenotypic variability for certain traits. An example of this is given in Figure 1, which illustrates the differences observed in young plants grown from seeds of a single self-pollinated plant derived from accession Hawaii 4. These natural variations suggest an underlying level of heterozygosity, and have the potential to add complexity to downstream genetic analyses or assessment of gene function, making it difficult to assess if phenotypes arise from a transgene, genetic lesion, or genetic variation. The variability we have seen in this line and in our parental Yellow Wonder line also complicated interpretation of physiological studies. For this reason, large numbers of plants were needed to achieve statistical significance when measuring parameters such as crown number, seedling root length and branching, or flowering time and number (J. Slovin, unpublished).

A solution to these problems would be to develop an easily grown, genetically uniform line with a documented homozygous genotype. Inbred lines can serve as standard genotypes for studies of gene action and biochemical pathways [21]. In our own laboratories, assessment of traits such as tolerance to abiotic stresses using measurements of root growth for example, would benefit from such a genetically and phenotypically homogeneous starting population. Documented inbred *Fragaria *lines will facilitate comparisons between experiments and among laboratories using T-DNA insertion mutants or overexpression studies to test gene function in strawberry, and perhaps more widely in the family Rosaceae.

The inbred line of *F. vesca *f. *semperflorens *var. Yellow Wonder described herein, YW5AF7, was developed at Beltsville, MD to facilitate such gene function studies in the genus *Fragaria*. We chose to start with commercially available seed called "Yellow Wonder" because these plants are day neutral, do not runner, and have yellow fruit color. These three traits have been analyzed genetically in *F. vesca *and shown to be encoded by recessive genes [22-24], and efforts were being made to clone the responsible genes [23,24]. Seed designated as "Yellow Wonder" is available from several commercial sources and is listed by the United States National Clonal Germplasm Repository http://www.ars.usda.gov/Main/docs.htm?docid=11324 as PI 551827. PI 551827 is listed as being of uncertain pedigree and not available commercially in the United States. "Yellow Wonder" obtained from the Burpee Seed Company was used in a study to identify the yellow fruit color locus [25]. The Yellow Wonder seed used in this project for generating YW5AF7 was part of the seed collection at the USDA in Beltsville, MD.

**Figure 2:**
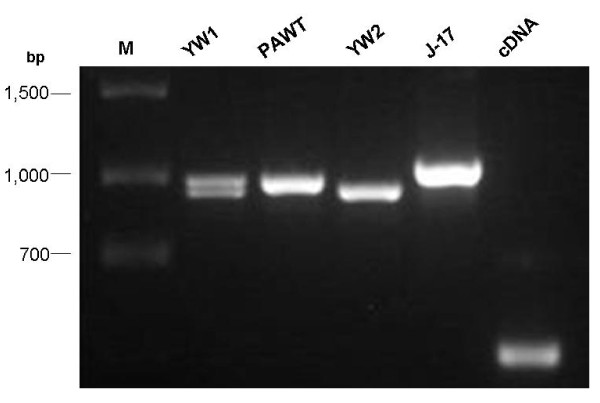
***F. vesca *lines called "Yellow Wonder" may not have the same genotype**. Amplification of a region of a gene encoding a mitochondrial low molecular weight heat shock protein shows differences between "Yellow Wonder" plants from two different sources (Y1 and Y2). Primers were designed to amplify a region containing an intron, and reveal polymorphisms between Y1 and Y2, as well as between subspecies of *F. vesca *(Y1, Y2 and Pawt) and between different diploid *Fragaria *species, *F. vesca *and *F. iinumae *(J-17). The same primers were used to amplify this region from a heat treated "Yellow Wonder" (Y2) seedling cDNA library (cDNA). bp: size markers in base pairs. M: size ladder.

The need for a standardized line that can be used by all laboratories for gene function studies with confidence in its genotype can clearly be seen in Figure 2, which shows a comparison of two lines designated "Yellow Wonder" from different sources. PCR products from the Burpee "Yellow Wonder" line (YW1) used by Deng and Davis [25] are clearly different from the product obtained with DNA from the "Yellow Wonder" line (YW2) used for generating YW5AF7. Also shown for comparison in this figure are the products obtained with DNA from a different *F. vesca *subspecies (Pawt), and a different diploid *Fragaria *species, *F. iinumae *(J-17). The region amplified is the intron in a mitochondrial low molecular weight heat shock protein identified from our heat-treated "Yellow Wonder" seedling cDNA library. With the resolution of one molecular character it is apparent that not all "Yellow Wonder" accessions are equivalent.

The advanced inbred diploid genotype, YW5AF7, provides a tool for direct tests of gene function in strawberry and other members of the Rosaceae family that can be used with confidence by all members of the research community. In this report we present information about the background of the accession, assessment of horticultural traits, and protocols for transformation and regeneration.

## Results

### Technical Description

#### Plants

**Figure 3:**
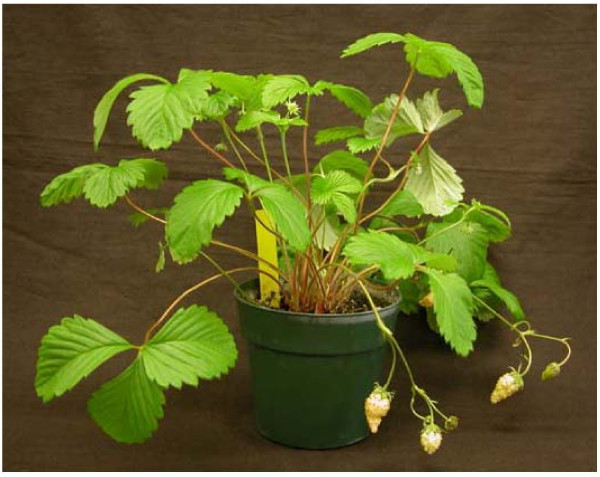
**The YW5AF7 plant**. The image shows a mature YW5AF7 plant with flowers and fruit in a 10 cm pot.

At 23°C, YW5AF7 seedlings in 10 cm. pots will flower by 8 weeks after sowing. Four weeks later the achenes can be harvested and sown to start the next generation, even though the berry they grew on may not be completely ripe and the achenes may be slightly green. By twenty weeks, numerous berries are present and ripe (Figure 3). Six-month-old greenhouse grown plants in 15 cm pots average 25 cm in height and can be 35 cm across. Plants produce large numbers of branch crowns and can fill a 15 cm pot within 8 months when supplied with fertilizer biweekly.

The leaves of YW5AF7 are thin, and show typical morphology for *F. vesca*. The leaf is light medium green in color, with both sides pubescent. Stomates are found on the abaxial side only. The first two true leaves are unifoliate, round and serrated. Later leaves are trifoliate, although very small ectopic highly serrated unifoliate leaves are also sometimes found at the base of the plant. Under greenhouse conditions in a 15 cm pot, the largest leaves can reach 13 cm in width and 8 cm in length. The terminal leaflet is ovate and more rounded than found on *F. vesca *var. Ruegen grown under the same conditions. It is serrated, with an average of 19 serrations on the largest terminal leaflets, whereas the margins of the same size terminal leaflet of the octoploid *F*. ×*ananassa *var. Chandler has about 25 serrations. Lateral leaflets of the largest leaves of YW5AF7 average 17 serrations. Serrations begin about half way up the inner edge of the lateral leaflets. The interveinal lamina are crinkled. Petioles are long and have a distinct adaxial groove. They are red in low light and tend to be greener toward the leaf. Petioles are pubescent, with straight, unbranched hairs. Stipules are red.

Flowers and fruit are borne within the leaf canopy as well as on inflorescences that extend above the canopy. Occasionally these extend down over the sides of the pots because of the weight of the fruit. In 15 cm pots, pedicels of the first inflorescence can be 15-20 cm long. Pedicels are round, pubescent, and tend to be greener than the petioles. Inflorescences usually have 4 to 5 flowers, however, under some conditions the cymous inflorescence continues to branch and form additional flowers. Root initials sometimes form at the nodes and these will form roots if pegged to the soil. On very old, pot-bound plants, inflorescences with only one or two flowers become common.

The flower is also typical of *F. vesca*. It usually has five petals, a calyx consisting of 2 whorls of five sepals, and 20 stamens. In the center of the flower is a rounded receptacle, bearing yellow pistils, that extends well beyond the stamens when the flower bud opens and the anthers dehisce. Occasionally flowers have extra flower parts, the most obvious of these occurrences being 6 petals per flower. In addition, petaloid anthers have been observed. Both conditions are also seen in the parental generations, and the appearance is correlated with larger flowers. Primary flowers are usually 1.5-2 cm in diameter, depending on growth conditions. Secondary and later flowers tend to be smaller.

#### Fruit

Like its progenitor, Yellow Wonder, the berry of YW5AF7 is soft and pale yellow in color with tan achenes when ripe, and pale green to white during development. Ripe berries are highly aromatic with sweet banana and pineapple overtones. When all achenes are fertilized, the berry shape is long conic, with some primary berries being necked long conic and reaching 27 mm in length and 20 mm in width in plants growing in 15 cm pots. The average fresh weight of a primary berry was 1.67 ± 0.26 S.E. g., with the largest berry being 2.45 g. Achenes are borne on the surface of the berry, with an average 193 ± 17 S.E. achenes per primary berry (225 on the largest). In comparison, an average of 518 achenes per primary fruit was reported for a commercial octoploid variety [26]. In the absence of insects or human interventions, only about half of the achenes are fertilized and enlarge, and the fruit tend to be smaller and of varied shapes. Pollination can be aided by transferring pollen from a flower with dehiscing anthers to a just opened flower using a small camel hair brush. The extent of fertilized ovules per fruit can be approximated over time by examining the expansion of developing achenes and the subtending receptacle tissue.

### Performance

Seeds of YW5AF7 will germinate in soil in the greenhouse in one week. However, more uniform germination can be achieved by cold treatment of moist seed. Seeds of YW5AF7 in moistened soil did not achieve maximum germination [87%, n = 100 (10 pots with 10 seed each)] until 21 days after sowing. Following treatment of moist seed for 3 weeks in the dark at 5°C, 74% of YW5AF7 seeds germinated in 7 days after being brought into the greenhouse, and by 14 days, maximum germination, 91%, was achieved. Seeds that have been disinfested using ethanol and bleach treatment will germinate in Petri dishes on 0.5× MS media [27] solidified with 0.8% Phytagar (Invitrogen, Carlsbad, CA). The resulting seedlings can be used as aseptic explants for tissue culture. Under these conditions we have found that longer cold treatment (>3 weeks) results in more uniform germination, which can be critical for evaluating developmental or physiological parameters of transformed plants in gene function tests.

Mildew susceptibility in *F. vesca *was found to be due to two dominant genes [28]. Both genes had to be absent to obtain a resistant plant, and cytoplasmic effects were noted. Seedlings of YW5AF7 are susceptible to powdery mildew at early stages in development, particularly in a growth chamber environment. In the greenhouse, YW5AF7 has been found to be susceptible to powdery mildew, thrips, two spotted mites, and aphids.

### Transformation and Regeneration

While YW5AF7 is a strong candidate for genomics studies, it was important to test if it could be successfully transformed and regenerated. While diploid strawberry is routinely transformed, transformation and regeneration efficiency are highly genotype specific [reviewed in [29]]. To test the YW5AF7 line for the ability to produce shoots after gene insertion, explants from greenhouse-grown YW5AF7 plants were co-cultivated with *Agrobacterium tumefaciens *carrying a visible GFP reporter as described in Materials and Methods. Several published media formulations were evaluated for regeneration and are detailed in Table 1.

**Table 1:** The media formulations used in regeneration experiments.

Medium	Auxin	Concentration	Cytokinin	Concentration	Reference
A	IBA	0.98 uM	BA	13.20 uM	[32]
B	2,4-D	0.45 uM	TDZ	4.54 uM	[36]
C	IBA	1.50 uM	TDZ	10.00 uM	[30]

**Figure 4:**
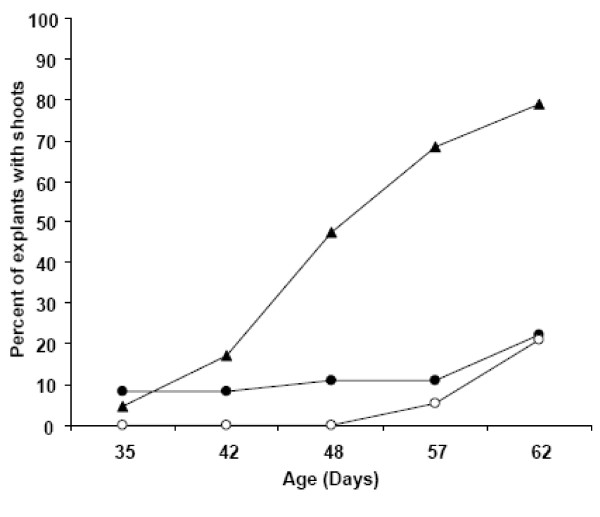
**YW5AF7 regeneration frequency on three published media formulations**. Various explants from YW5AF7, including mature leaves, young leaves and petioles, were grown on three different media (Table 1) to test regeneration frequency. Medium A, white circles; Medium B, black circles and Medium C, triangles. The data are the means of two independent experiments.

**Figure 5:**
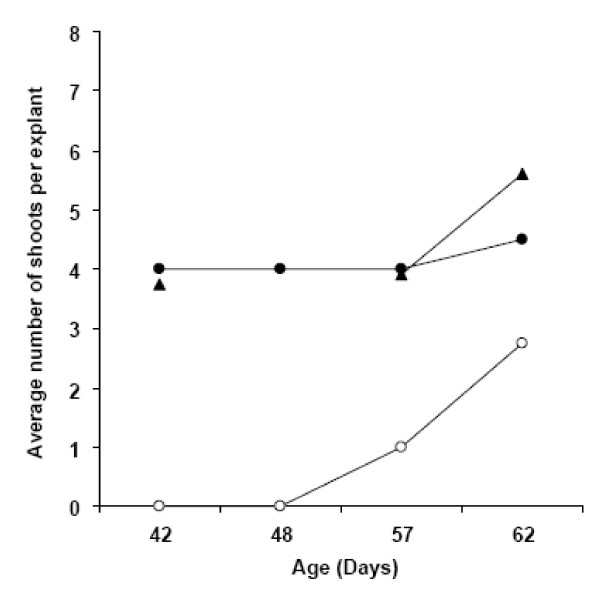
**The number of shoots per YW5AF7 explant on three media formulations**. The mean number of shoots per explant was determined for three different media: Medium A, white circles, Medium B, black circles and Medium C, triangles. The data reflect the mean of two independent experiments.

Callusing was observed on all media types tested, but tissue vigor and regeneration were best supported by the formulation presented in Zhao et al. [30] (Figure 4, triangles). On this formulation, 20% of explants possessed shoots by six weeks whereas explants on other media formulations exhibited little or no organogenesis at this time point (Figure 4, black or white circles). At nine weeks, over 80 percent of explants had shoots on the Zhao formula, whereas shoot initiation on other media types was less frequent. Figure 5 shows that the medium producing the highest percentage of explants exhibiting organogenesis also resulted in a higher number of shoots per explant by nine weeks in culture than other formulations.

**Figure 6:**
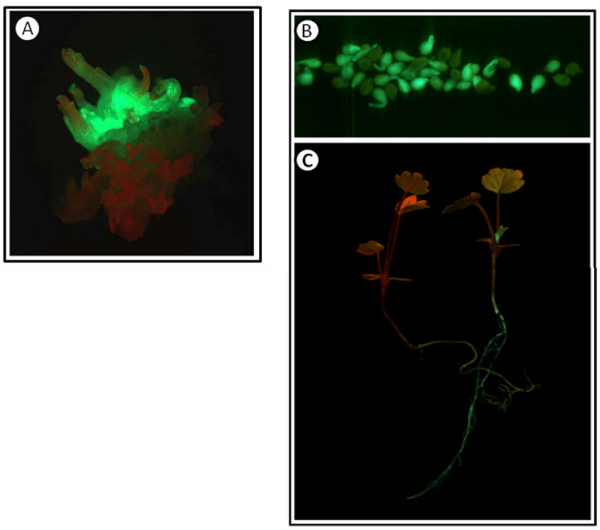
**YW5AF7 transformants**. A. A cluster of shoots emerging from the basipetal end of a petiole. Both transformed (GFP+; green) and non-transformed shoots (red in color) are present. B. Imbibed seed from one line of GFP expressing transformed 5AF7 plants. GFP positive and GFP negative seeds are present in a 19:20 ratio, indicating that a single insertion is likely. C. A wild-type seedling (left) and a GFP positive transgenic seedling (right) grown from seed of the plant in (B).

These data indicate that the formulation by Zhao et al. [30] results in the highest number of explants exhibiting shoots, which is important for maximizing the number of independent transformation events in gene function experiments. Shoots were generated by direct organogenesis and were produced most quickly and abundantly on the basipetal end of petiole segments (as shown in Figure 6A). Evaluation of GFP fluorescence in emerging shoots revealed that about 40% of shoots were transformed, indicating that escapes can be present using 4 mg/L hygromycin for selection.

Once differentiated, explants were moved to a media without TDZ to enhance shoot elongation. Although not formally quantified, at least one insertion event was observed on each explant, as evidenced by GFP fluorescence. Figure 6 shows germinating seed and two resulting seedlings from one such plant. In this random sample of seeds, the ratio of GFP positive to GFP negative (wild type) seeds was essentially 1:1, indicating that there was most likely a single insertion event in this transformant.

## Discussion

*F. vesca *has great potential as a system to study the genetic basis of agriculturally important biological questions in the Rosaceae family. Its small size, rapid growth, generous seed set, small genome, and sequence availability make it an excellent resource for development of genomics tools. Its genomic similarity to other valuable crops underlies its potential utility as a surrogate to test gene function relevant to many rosaceous species.

A primary concern about the system has been the observation of variability among individuals in lines that have not been single seed propagated in the lab through several generations. Although typically self fertilizing and therefore expected to be largely homozygous, we observed clear variability in a number of horticultural traits among *F. vesca *Yellow Wonder and Hawaii-4 plants generated from achenes from a single fruit suggestive of some degree of residual heterozygosity. These phenotypic variations are potentially problematic in a seminal line proposed as a genomics-friendly genotype. This is an important consideration as several current efforts are developing populations of T-DNA insertion, activation tagged, overexpression, or RNAi lines using *F. vesca*, and it could become difficult to discriminate between a phenotype resulting from an engineered genotypic variation and natural genetic variation in the line. Interpretations from a genetically noisy background may preclude, or at least delay, identification of gene-specific effects on morphology and physiology. High variability in results from physiological studies drastically increases the number of plants that must be used to obtain statistical significance. These populations may be suboptimal for quantitative studies of gene expression, as the variation in the baseline may lead to errors in interpreting microarray, digital, or qRT-PCR gene expression profiles. Results must be able to be repeated in other laboratories, so a system based on a known genetic background will supplement these efforts and be of benefit to the wider research community.

Our PCR analysis of two different "Yellow Wonder" lines (Figure 2) indicated that even a well-established and commercially available line of *F. vesca *may consist of different genotypes. Because "Yellow Wonder" is both non-red and non-runnering it would appear likely, given that these two loci must be homozygous recessive, that these plants are already substantially homozygous. However, clearly Y1 is not the same as Y2. There are no data from any of the suppliers to show that their "Yellow Wonder", the color of which would be expected to breed true from seed, is the same as a competitor's, which also would breed true from seed (at least for color and non-runnering), and no data to show that any of these are the same as others described in the literature. For these reasons, the pre-emptive development of a stable, highly inbred, prolific and documented genotype was considered useful, as it would provide a stable genotype for evaluation of gene function that could be shared among users.

Botanical descriptors of YW5AF7 have been carefully evaluated and define a reproducible and firm foundation for later comparisons. Even subtle phenotypes induced by a transgene should be able to be reliably scored in this stable background.

Many of the techniques used for studying *Arabidopsis *can be used with *F. vesca *YW5AF7. Seedling variations are almost indiscernible in populations of Arabidopsis seedlings, and their small stature makes *in vitro *assessment of phenotypes possible. Tests of early development in response to environmental conditions, growth regulators or nutrient status are also possible in *F. vesca*, much like in *Arabidopsis*.

For YW5AF7 to have utility as a functional genomics system it must be transformable. As observed by many groups, transformation efficiency of various strawberry genotypes is highly genotype dependent and in some cases impossible [29]. The transformation capacity of YW5AF7 was tested with a GFP reporter gene. Many GFP foci were observed in co-cultivated tissues and GFP-positive plantlets were regenerated on media containing selective antibiotics. Three published (yet diverse) media formulations were evaluated for regeneration-inducing ability. In all cases shoots appeared via organogenesis with the best results arising from the media formulation presented in Zhao et al. [30]. A number of shoots were clearly initiated by 30 days and plantlets could be excised after 60 days and rooted in rooting media. This time course is reasonable yet could likely be optimized to improve the utility of the YW5AF7 system. The high frequency of shoot formation on independent explants ensures propagation of independent transformants. Antibiotic sensitivity was generally consistent with previously-published reports in strawberry [31,32] but subculture to progressively higher amounts of antibiotics may be advisable as regeneration of non-transformed shoots was observed using hygromycin at 4 ug/ml.

The most prolific explants were leaf-adjacent petiole segments, with the first shoots appearing on the basipetal end of these explants. The most productive formulation contained thidiazuron, TDZ, as a principle growth regulator, a compound shown to be effective in inducing regeneration in a number of other studies. However, consistent with previous reports [33] growth on TDZ severely stunted shoot elongation, and increased somaclonal variation has been observed when this regulator has been employed [see, [34]]. Once clearly formed, the shooting explants were transferred to a TDZ-free media formulation that had been used to regenerate *F. vesca *accession Hawaii-4. Within one week the shoots elongated vigorously and could be transferred to rooting media. Other media formulations also induced shoots, but at a much slower rate.

The YW5AF7 line is runnerless and this has distinct advantages to its adoption as a functional genomics model. Runnerless plants are much easier to maintain in a greenhouse as plants may be located in close proximity without having to continually remove a tangle of runners or daughter plants that invade neighboring pots, such as is our experience with *F. vesca *Hawaii 4 and *F. vesca *Pawtuckaway. In large populations this can be a source of genotype contamination and requires dedication to constant manual management. On the other hand, one of the advantages of *F. vesca *as a functional genomics system is that plants can be propagated by branch crown divisions or runners or branch crown division, as well as by seed, making it possible to vegetatively propagate mutants that affect flowering or seed set. Although runnerless, the YW5AF7 line does produce abundant branch crowns.

Seeds of YW5AF7 are available for research purposes from Dr. J. P. Slovin, USDA-ARS Genetic Improvement of Fruits and Vegetables Laboratory, Bldg. 010A, 10300 Baltimore Avenue, Beltsville, MD 20705 (phone: 301/504-5629; e-mail: slovinj@ars.usda.gov). The seed are produced in the greenhouse under ambient conditions from F7 plants that are manually self-pollinated. Seed of this line have been deposited with the USDA, ARS National Clonal Germplasm Repository, Corvallis, OR (PI 641092).

## Conclusion

The highly inbred selection YW5AF7 has been generated and characterized. A set of botanical descriptors defines a baseline that may be compared to phenotypes of forward or reverse genetic mutants as well as overexpression and RNAi lines. This genotype, associated scored metrics, and transformation protocol permit the deployment of this system as a useful tool for the Rosaceae research community in the elucidation of gene function in a stable and consistent genetic background that complements existing systems.

## Methods

### Plant Origin and Seedling Selection

The inbred line, designated YW5AF7, was obtained by manually self-pollinating *F. vesca *var. "Yellow Wonder" plants grown from seeds in the Beltsville collection maintained originally by S. Hokanson. Seeds were planted in Metro Mix 510 (Scotts-Sierra Horticultural Products, Marysville, OH) supplemented with dolomitic lime. Plants were grown in the greenhouse with supplemental lighting from sodium halide lamps to give a daylength of at least 12 h. At least one hundred seed were planted at each generation. Flowers were self-pollinated by gentle brushing starting when their flowers just opened, and every day thereafter as their anthers dehisced until the flower petals fell. A new small camel hair artist's brush was used for each pollination. Flowers were tagged for the day of pollination. The plant to be used for the next generation was randomly chosen from ten plants that met a basic requirement of early flowering, general robust appearance, fruit set (as based on number of achenes that enlarge following pollination), high number of achenes on the primary fruit, and short number of days to mature fruit. Although germination rate and number of days to flowering varied, the other selecting parameters usually showed very little difference. However, some seedlings germinated from F3 seed showed morphological abnormalities such as dwarfness or a single cotyledon. Only seedlings with normal phenotypes were selected for further selfing. The selfing process was continued for seven generations to generate YW5AF7.

### Media Preparation

Three previously defined media formulations were investigated to determine which would lead to optimal regeneration of YW5AF7 explants. Media included 1× Murashige and Skoog medium with vitamins, 2% sucrose and the growth regulators presented in Table 1. Media were prepared with deionized water, the pH adjusted to 5.6-5.8, and then autoclaved for 20 min at 121°C and 15 psi. Growth regulators were co-autoclaved with the media. In all three media types, the selection agent used was 4 mg/L hygromycin B, which was added to the media after it was cooled to ~50°C.

### Agrobacterium-mediated transformation

Leaf, stem, and petiole segments were sterilized in 70% EtOH for 30 seconds and 1% sodium hypochlorite (20% bleach) for 10 min. A single transformed *Agrobacterium *colony carrying a 35S::GFP construct was grown overnight in Luria Broth with 10 mg/L rifampicin, 50 mg/L gentamicin, and 50 mg/L spectinomycin to an OD_600 _of 0.5, then pelleted at 1,000 × g. The bacterial pellet was resuspended to 0.1 OD_600 _in co-cultivation medium consisting of 1× MS pH 5.8 with 2% sucrose, supplemented with 50 uM acetosyringone. Explants were added to the co-cultivation medium and incubated 20 min at room temperature, then blotted dry with sterile filter paper and transferred to media without selection for 2 d at 25°C in darkness. After 2 d the explants were washed twice in co-cultivation medium liquid supplemented with 500 mg/L carbenicillin, followed by 30 min of incubation in fresh wash media. Explants were again blotted dry and transferred to solid media with selection.

Transgenic shoots were selected on media containing 4 mg/L hygromycin B. The explants were regenerated under a 16 h light, 8 h dark photoperiod under cool-white fluorescent lighting. Explants were checked daily for contamination, and subcultured every 2 weeks. When distinct clumps of shoots were formed the entire clump was transferred to hormone-free rooting media consisting of 0.5× MS media (pH 5.8), 1% glucose, and 1% phytoagar. Roots formed within days to one month and individual plants could then be dissected from the groups of shoots.

### PCR

PCR was performed using the touchdown protocol described by Sargent et al. [35] in a 20 μl reaction containing HotStart Taq Master Mix (Qiagen, Valencia, CA), 0.4 μM each primer, and 1.0 ng genomic DNA. PCR products were separated by electrophoresis through a 1.5% TAE agarose gel and visualized by ethidium bromide staining. Primers were designed to span the intron in the N-terminal domain of a low molecular weight heat shock protein gene identified as an EST (GenBank accession number CX661743.1) from a "Yellow Wonder" (Y2) heat-treated seedling cDNA library. Template DNA for Y1, Y2, *F. iinumae *J-17, and *F. vesca *subsp. *americana *Pawtuckaway was obtained from 50-100 mg young leaf tissue using a DNeasy Plant Mini kit (Qiagen). Y1 and *F. vesca *subsp. *americana *Pawtuckaway plants were obtained from T. Davis (University of New Hampshire) and *F. iinumae *J-17 plants were obtained from the US National Plant Germplasm collection.

## Competing interests

The authors declare that they have no competing interests.

## Authors' contributions

JPS conceived of the project, performed all crosses, plant measurements and PCR, and participated in preparation of the manuscript. KS performed the co-cultivation and subculturing of strawberry tissues to test transformation efficiency. KMF supervised transformation and regeneration and participated in preparation of the manuscript.
